# A Rural "Sanitary" District

**Published:** 1906-02-03

**Authors:** 


### The Hospital.

A Journal of

The Medical Sciences and Hospital Administration.

Vol. XXXIX.—No. 1,010. SATURDAY, FEBRUARY 3, 1906.

### A Rural "Sanitary" District.

We could not foresee, when writing last week of the state of Metropolitan sanitation and of the opportunities which it is calculated to afford to a reforming President of the Local Government Board, that the Board itself was on the point of publishing evidence to prove that such opportunities are still more abundant in rural districts, and that the sufferings of the inhabitants of those districts, from purely preventible evils which it would be within the power, as it is within the duty, of "local authorities" to remove, are such as to form a very considerable factor of the conditions tending towards national physical degeneration. The Board, without waiting for the time at which the next annual report of its medical officer will be given to the public, has issued one of the appendices of that report in anticipation of the whole—an appendix containing the account given by Dr. Farrar of his investigation of the "sanitary circumstances and administration of the Clun Rural District, Salop." It appears that this district is governed by a "Rural Council," and that it comprises two "divisions"—Bishop's Castle and Clun. Until April 1904 the Council had employed a person who was called an inspector of nuisances for each division; but, at that date, they proposed to combine in one person the offices of relieving officer and inspector of nuisances for the Bishop's Castle division. As it is contrary to the practice of the Local Government Board to assent to this combination of offices, Dr. Farrar was instructed to undertake a complete inquiry into the circumstances of the case.

Taking his narrative as a whole, it is perhaps best described as furnishing a remarkable example of the extent to which the beneficent operation of the laws of Nature will defeat the endeavours of mankind to cultivate disease by an aggregation of many of its most formidable predisposing causes. The report contains no record of the sickness or mortality of the district, and it may be presumed that the bacilli of enteric fever, of diphtheria, or of analogous forms of disease, had by some good fortune not been brought within its boundaries. In various hamlets there are instances of stables, cow-houses, or pigsties, built against the outer walls of dwelling rooms, these walls being of such structure as to favour percolation into the dwelling. In a number of cases the waste-pipe of a slop-sink opens directly into the house-drain. The natural water supply is good and plentiful, but it is subject to every variety of pollution before it reaches the inhabitants. Less than half the population of the district derive their water from supplies conveyed by pipes; the remainder draw water from shallow wells, from springs (sometimes converted into dip-wells), or from brooks. In too many cases the supplies thus obtained are inadequate in amount or are liable to serious pollution. The neighbourhood of the wells is often fouled and trampled by cattle; many of them are so placed as inevitably to receive soakage from ill-paved stables or foldyards, and Dr. Farrar mentions one case in which the mouth of the shallow well is situate on a ledge of the hillside. On a level five feet below this ledge is the ill-paved floor of a pigsty, and in the angle between the floor and the wall of the ledge he observed, standing in a hole where the pavement was defective, a large stagnant pool, within six feet of the side of the well, and so placed as to drain into it. The drainage of villages in the district is "haphazard and unsystematic," sewage finding its way into water-courses and roadside gutters. In nearly every farm the foldyard is unpaved, and constitutes a quagmire of rotting manure, the drainage of which runs away through the gate into the public road. This state of things is perhaps worst in the village of Brocton, and the condition of many farmyards in the county has lately been the subject of a report to the County Council by their chief veterinary officer in connection with the spread of anthrax. The small town of Clun itself has a system of drainage, but its sewage is discharged untreated into the river of the same name; and the closet accommodation of the schools of the district is indescribably filthy. It is hardly necessary to add that there is no provision for the isolation of infectious disease, no disinfector, and that one "registered" cowshed in Clun was "very filthy, paved with defective cobbles, and having no proper fall for drainage; the shed was ill-ventilated, and a large heap of manure was piled against the outside of the wooden wall."

Such, greatly condensed, is the official description of the state of things permitted to exist in rural

England by the local authorities to whose care the health of the population is committed. Of the present inspectors of nuisances one "keeps a small fishmonger's and grocer's shop, and, travelling the country for orders with his cart, combines this business with his inspectorial duties"—an arrangement which Dr. Farrar describes as "not conducive to the efficient or impartial discharge of official obligations." The other inspector is the relieving officer whose appointment in the double capacity was the immediate cause of the inquiry; and neither of these officers holds the certificate of the Sanitary Inspectors' Examination Board, or any other certificate in sanitary science. Is it not the imperative duty of the Legislature to protect the humble folk of villages against such ignorance and such mal-administration as the report from which we have been quoting officially describes?

